# Homeostatic properties of *Lactobacillus jensenii* engineered as a live vaginal anti-HIV microbicide

**DOI:** 10.1186/1471-2180-13-4

**Published:** 2013-01-08

**Authors:** Hidemi S Yamamoto, Qiang Xu, Raina N Fichorova

**Affiliations:** 1 Laboratory of Genital Tract Biology, Department of Obstetrics, Gynecology and Reproductive Biology, Brigham and Women’s Hospital, Harvard Medical School, 221 Longwood Avenue, Boston, MA, USA; 2 Osel, 320 Logue Avenue, Mountain View, CA, USA

## Abstract

**Background:**

Vaginal probiotics are investigated as a binary strategy for prevention of bacterial vaginosis and HIV. We applied an innovative experimental model using primary and immortalized human cervical and vaginal epithelial cells to assess the functional properties of *Lactobacillus jensenii*, a predominant constituent of the healthy vaginal microbiome, engineered to express the HIV-1 entry inhibitor modified cyanovirin-N (mCV-N). In this model bacteria colonize the epithelial cells over a period of 24-72 h. Staurosporine and the Toll-like receptor 2/6 ligand macrophage-activating lipopeptide-2 (MALP-2) serve as positive controls for apoptosis and proinflammatory activation, respectively. In 24-hour intervals, the colonized epithelium is assessed microscopically, supernatants are collected for measurement of soluble immunoinflammatory mediators and production of CV-N, and cells are lysed for assessment of: 1) apoptosis by cleaved versus total caspase-3 assay; 2) NF-κB activation by a luciferase reporter assay; or 3) epithelia-associated colony forming units (CFU) in Brucella agar.

**Results:**

Wild type (WT) *L. jensenii* 1153 consistently colonized cervical and vaginal cells in the absence of epithelial damage and apoptosis. The bioengineered derivatives expressing mCV-N or control plasmids showed the same stable colonization pattern, which was reproducible between technologists and bacterial batches (CFU coefficient of variation <10% within and between experiments and epithelial cell types). MALP-2 activated NF-κB and caused fold-increased levels of proinflammatory mediators with clinically established significance in the cervicovaginal environment (IL-1α, IL-1β, IL-6, TNF-α, IL-8, RANTES, MIP-3α, and ICAM-1), measured by a multiplex electrochemiluminescence assay. At the same time levels of protective anti-inflammatory mediators interleukin 1 receptor antagonist (IL-1RA) and secretory leukocyte protease inhibitor (SLPI), both measured by ELISA, remained constant (IL-1RA) or moderately increased (SLPI). Similarly to MALP-2, colonization by *L. jensenii* WT activated NF-κB; however, unlike the synthetic TLR2/6 ligand, the live microorganisms did not induce significant changes in the secreted levels across all inflammation-associated proteins. The mCV-N production and function were confirmed by western blot and a HIV-1 gp120 binding assay, respectively. The bioengineered lactobacilli expressed mCV-N with anti-HIV activity preserved in the epithelial cell context and caused no significant immunoinflammatory changes as compared to the WT *L. jensenii*.

**Conclusions:**

These results highlight the translational value of the colonization model and justify further clinical investigation of the homeostatic and anti-HIV effectiveness of the *L. jensenii* derivates.

## Background

Topical microbicides have been investigated as a leading prevention strategy in the HIV/AIDS pandemic, which currently affects 34 million people around the globe [1]. A number of compounds with broad-spectrum anti-HIV activity *in-vitro* have successfully passed preclinical and Phase I evaluations, nevertheless, those selected for Phase II/III trials have failed to prevent HIV thus far [2-6]. Anti-retrovirals with more specific anti-HIV activities have also been explored; however, tenofovir, the only topical gel candidate tested in Phase II/III settings as of yet, had initially demonstrated marginal (39%) effectiveness [7], but has most recently been discontinued due to futility [8].

The impracticality and numerous pharmacokinetic difficulties of the coitally- related dosing strategy are shortcomings of the conventional gel-based microbicides [2,3,7,9,10]. Gels may not efficiently cover the entire genital tract mucosal surface vulnerable to HIV entry. Typically gels require application shortly before intercourse to be protective and frequently may require re-application to counter the effects of dilution, degradation or rapid clearance [11]. On the other hand, frequent exposure of the vaginal environment to foreign substances can have toxic effects and damage the epithelial membranes resulting in irritation and undesirable inflammatory responses increasing the risk of HIV acquisition [12]. A solution to these shortcomings may be offered by bioengineered probiotic products based on vaginal/rectal commensal organisms that are capable of delivering anti-HIV factors in a sustainable, non-inflammatory, self-renewing mechanism directly at the point of viral infection [13-19].

This study applied an innovative experimental model of microbiota colonized epithelium [20] to assess the immunoinflammatory properties of a probiotic-based anti-HIV microbicide. Osel, Inc (Mountain View, CA) has genetically engineered *Lactobacillus jensenii*, one of the predominant components of the normal vaginal microbiota [21,22], to express a modified version of the anti-HIV Cyanobacterium protein Cyanovirin-N (mCV-N) [15]. The natural CV-N protein interrupts HIV-1 membrane fusion by impairing CD4 independent and dependent binding of gp120 to the HIV-1 co-receptors CCR5 and CXCR4 [23,24]. Pusch *et al.* demonstrated HIV-1 inhibition *in-vitro* with another modified version of CV-N expressed by *L. plantarum* and *Lactococcus lactis*[16]. The bioengineered mCV-N invented by Osel Inc. irreversibly inactivates both CXCR4 and CCR5 tropic HIV strains *in-vitro*[15,23]. *L. jensenii* expressing mCV-N at concentrations of 7×10^8^ CFU/ml*,* mimicking the natural *L. jensenii* concentrations found in women [25], completely inhibited CCR5 tropic HIV-1 entry *in-vitro*[15,26]. Both the natural CV-N and mCV-N are inhibitory against T-tropic, M-tropic and dual T and M-tropic primary clinical strains of HIV-1 and T-tropic laboratory adapted strains of HIV-1 and HIV-2 *in-vitro*[15,23]. *L. jensenii* 1153 was selected as a parental strain due to it’s growth, colonization rates and inherent probiotic properties [15]. Our study is the first to assess simultaneously the colonization and immunomodulatory properties of 1153 and its mCV-N producing derivatives in the human vaginal epithelial cell context. Hereby we tested the hypotheses that: 1) an *in-vitro* model can mimic key components of the microbiota-epithelial interactions in a sustained reproducible manner allowing comparison of multiple bioengineered strains, 2) genetically engineered *L. jensenii* strains can deliver a bioactive anti-HIV peptide in the context of an unharmed homeostatic epithelial-commensal microenvironment.

## Methods

### Bacterial strains

The parental wild type (WT) *L. jensenii* 1153 human vaginal isolate and five experimental derivatives (Table 1) were obtained from Osel, Inc (Mountain View, CA). The generation of the bioengineered strains was previously published [15].

**Table 1:** **Bioengineered *L. jensenii *derivatives with the expression cassette stably integrated into the bacterial chromosome**

**Strain**	**Integration Site**	**Expression Cassette**	
		**Promoter**	**Integrated gene**
*L. jensenii* 1153^a^	NA^b^	NA	NA
*L. jensenii* 1153-1666	*pox1*	*rpsU*	*APVT*-CV-N (P51G)
*L. jensenii* 1153-2666	*pox1*	*ptsH*	*APVT*-CV-N (P51G)
*L. jensenii* 1153-3666	*pepO*	*rpsu*	*APVT*-CV-N (P51G)
*L. jensenii* 1153-1646	*pox1*	*gusA*	Gus A (β-glucoronidase)
*L. jensenii* 1153-GFP	*pox1*	*rpsU*	EGFP^c^

^a^Parental *L. jensenii* strain^; b^NA=not applicable (wild type strain); ^c^enhanced green fluorescent protein.

### Control test agents

The synthetic macrophage-activating lipopeptide-2 (MALP-2) (Alexis Biologicals, San Diego, CA), a known Toll-like receptor (TLR) 2/6 ligand, was used at 50 nM as a pro-inflammatory control [20,27]. Staurosporine (Sigma-Aldrich, St. Louis, MO) was used at 1 μM as a pro-apoptotic agent [20,28,29].

### Epithelial models

Human immortalized endocervical (End1/E6E7) and vaginal (Vk2/E6E7) epithelial cell lines were grown in antibiotic-free keratinocyte serum-free medium (KSFM) (Invitrogen, Carlsbad, CA) supplemented with bovine pituitary extract, epidermal growth factor and calcium chloride as described [30]. These immortalized cell lines have been previously shown to closely resemble the columnar (End1/E6E7) and stratified squamous (Vk2/E6E7) epithelial differentiation patterns and immune responses of primary cells and normal tissues of origin [30-36]. Polarized tissue constructs VEC-100™ derived from primary ectocervical/vaginal epithelial cells, previously depicted immune properties comparable to that of normal tissues of origin [37,38] were purchased from MatTek Corporation, Ashland, MA. The VEC-100™ tissues were maintained in antibiotic-free medium provided by MatTek.

### Recovery of cryopreserved wild type bacteria and bioengineered derivatives

Multiple aliquots from three separate batches of *L. jensenii* WT and derivatives were received frozen from Osel, Inc and stored at −80°C until tested. Each batch was examined in a minimum of three independent experiments. All strains were tested simultaneously by comparison of colony forming units (CFU) before use in our epithelial colonization model. For that purpose, one aliquot per strain from each batch was thawed, washed once in PBS by centrifugation, serially diluted in PBS and plated onto Brucella-based agar plates (PML Microbiologicals, Wilsonville, OR). Plates were incubated in an anaerobic chamber (Coy Laboratory Products Inc., Grass Lake, MI) containing an atmosphere of 10% carbon dioxide, 10% hydrogen, 80% nitrogen at 37°C for 24 h-48 h (until visible colonies formed), followed by CFU counting. Percent recovery of viable bacteria was determined in comparison to CFU counts obtained prior to cryopreservation by Osel, Inc.

### Epithelial colonization

*L. jensenii* suspensions were prepared in antibiotic-free KSFM (Invitrogen) at 7×10^6^ CFU/ml to colonize epithelial surfaces for 24 h, 48 h and 72 h as previously described for other vaginal bacteria [20]. In the immortalized cell line model, epithelial monolayers were grown to 100% confluence in 96-well plates (Fisher Scientific, Pittsburgh, PA) and bacterial suspensions (0.1 ml) were added to achieve a multiplicity of infection of ~10:1. In the VEC-100**™** model, tissue inserts were placed over 0.5 ml medium in 12-well plates (Fisher Scientific) followed by addition of 0.156 ml bacterial suspension to the apical epithelial surface. The bacterial-epithelial cocultures were incubated for 24 h-72 h under anaerobic conditions generated by AnaeroPack System (Mitsubishi Gas Chemical Co. Inc., New York, NY), at 35°C on an orbital shaker. Cell culture supernatants from the immortalized epithelia and basal chamber culture fluids from the VEC-100 tissue model were collected in 24 h time intervals for measurement of soluble immune mediator levels and mCV-N as described below. At the end of each 24 h period the cells/tissue were washed and used for enumeration of epithelia-associated CFU (see below), or medium was reapplied and cultures were returned to anaerobic chamber for additional 24 h incubations. In some experiments, the cells were lysed for assessment of NF-κB activation or apoptosis (see sections below).

### Transmission electron microscopy

Vk2/E6E7 cells were seeded on Aclar embedding film (Ted Pella Inc. Redding CA) and colonized with *L. jensenii* strains for 24 h. A TecnaiG2 Spirit BioTWIN transmission electron microscope (FEI Company, Hillsboro, OR) was used to visualize bacterial-epithelial colonization, confirm morphological integrity and a lack of apoptosis as previously described [20].

### Epithelium-associated CFU enumeration

Association of viable lactobacilli with epithelial cells was assessed by CFU counts as described in detail elsewhere [20]. In brief, at the end of each time period, the cultures were washed twice with ice-cold PBS and hypotonically lysed for 15 min in ice-cold HyPure water (Fisher Scientific), followed by adjustment of osmolarity with 2× concentrated PBS (Invitrogen). Serial dilutions were prepared in PBS and 30 μl of each dilution was inoculated on Brucella-based agar plates (PML Microbiologicals). The plates were incubated in an anaerobic chamber (Coy Laboratory Products Inc) containing an atmosphere of 10% hydrogen, 10% carbon dioxide and 80% nitrogen at 37°C for 24 h-48 h (until visible colonies were formed), followed by CFU counting. CFU per cm^2^ epithelial surface area were calculated.

### NF-κB activation luciferase reporter assay

Endocervial epithelial cells stably transfected with pHTS-NF-κB firefly luciferase reporter vector (Biomyx Technology, San Diego, CA) as described [34] were grown in 96-well plates in hygromycin selection medium until confluence and then colonized with *L. jensenii* strains as described above. After 24 h, supernatants were collected, cells were lysed with GloLysis buffer and luciferase activity was determined using the Bright-Glo Luciferase Assay System by manufacturer’s protocol (Promega, Madison, WI).

### Caspase-3 assay

Vaginal epithelial cells (Vk2/E6E7) were treated with bacteria, MALP-2 (50 nM) and the proapoptotic agent staurosporine (1 μM) to serve as a positive control. At the end of each incubation period, the epithelial monolayers were lysed in Tris lysis buffer containing protease inhibitor cocktail provided by Mesoscale Discovery (MSD), Gaithersburg, MD, per manufacturer’s protocol. Levels of cleaved and total caspase-3 were measured simultaneously in each cell lysates using an MSD electrochemiluminescence (ECL) mutliplex assay and Sector Imager 2400 with Workbench software (MSD).

### Soluble immune mediators assays

Concentrations of interleukin (IL-1α, IL-1β, IL-6, TNF-α, IL-8, RANTES, MIP-3α, and ICAM-1) were measured in cell culture supernatants simultaneously using an MSD multiplex assay, Sector Imager 2400, and Workbench software. Levels of IL-1 receptor antagonist (IL-1RA) and the antimicrobial peptide secretory leukocyte protease inhibitor (SLPI) were measured by Quantikine ELISA (R&D Systems, Minneapolis, MN) using a Victor^2^ reader (Perkin Elmer Life Sciences, Boston, MA).

### mCV-N detection and functional recovery

Cell culture supernatants collected from the vaginal and cervical colonization models were sterilized through 0.2 micron PharmAssure’s Low protein binding syringe filters with HT Tuffryn Membrane (Pall Corporation, Port Washington, NY)*.* Western blot analysis of the filtered supernatants was performed as described [13] to ensure full length expression of CV-N in the experimental model, and to rule out loss of protein to filtration. The filtered sterile supernatants were subjected to a gp120 binding assay to confirm the presence of functional mCV-N in the epithelial context. In brief, 96-well plates (Aalto Bio, Dublin, Ireland) coated with anti-HIV-1 gp120 antibody bound to recombinant gp120 (Protein Sciences, Meriden, CT) were incubated with undiluted cell culture supernatants for 2 h to allow for gp120 binding. Bound molecules were detected by rabbit anti-mCV-N and anti-rabbit horseradish peroxidase (HRP) (Alpha Diagnostics, San Antonio, TX) as described [13].

### Statistical analysis

One-way ANOVA with Bonferroni multiple comparisons analysis were performed using GraphPad Prism version 4.00 for Windows (GraphPad Software, San Diego CA). *P* values <0.05 were considered significant.

## Results

### *L. jensenii* reproducibly and consistently associates with the primary and immortalized cervicovaginal epithelial cells in the absence of apoptosis

**Figure 1:**
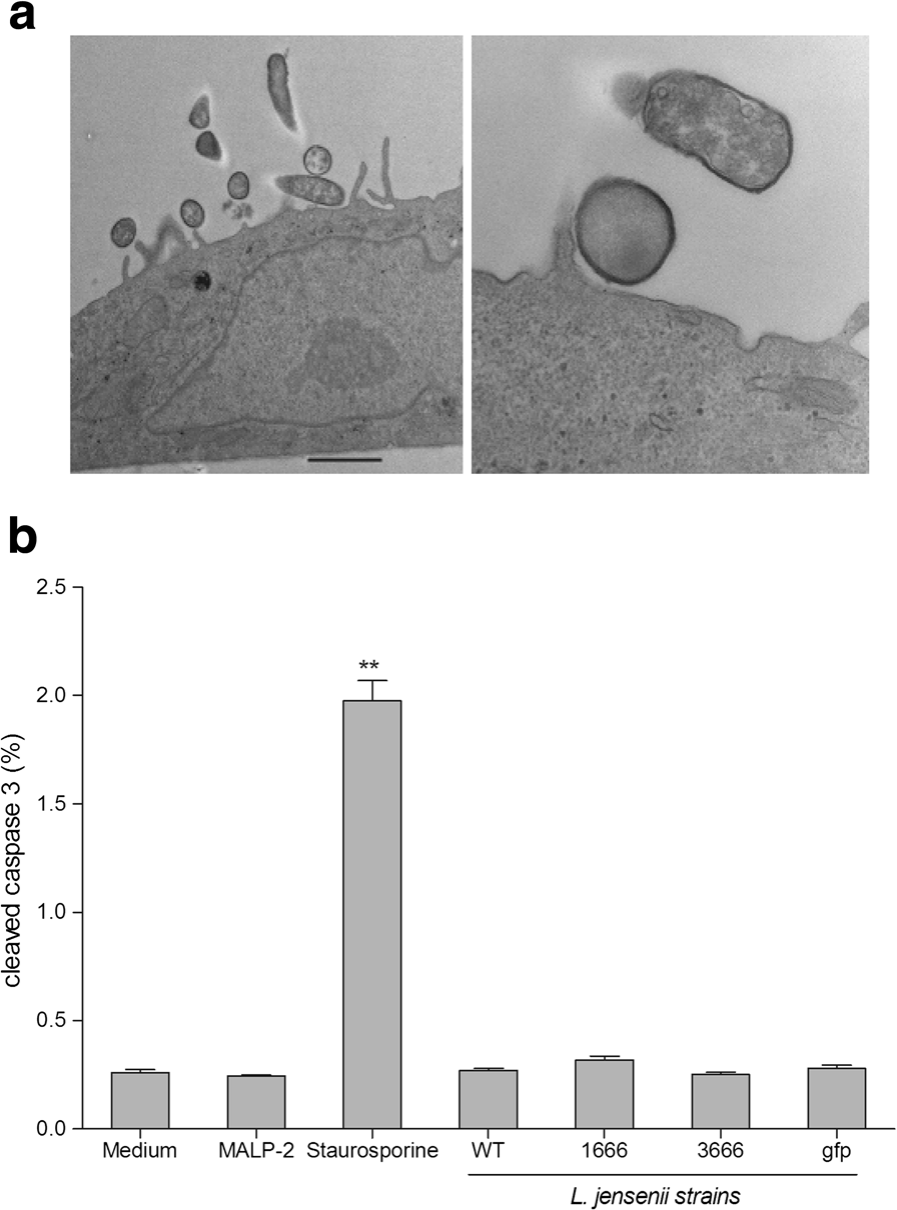
***Lactobacillus *strains consistently associate with the human epithelial model in the absence of apoptosis.** (Figure 1a) Transmission electron microscopic image illustrates clear association between the *L. jensenii* electron dense bodies and the morphologically intact vaginal epithelial cells. No morphological signs of apoptosis are present. Bar represents 2 microns with a magnification of x 4800. (Figure 1b) Caspase-3 cleavage represented by % cleaved over total caspase harvested from vaginal (Vk2/E6E7) epithelial lysates after 24 h colonization with *L. jensenii* 1153 wild type (WT) and bioengineered *L. jensenii* 1153–1666, 3666 and gfp strains or treatment with 1 μM Staurosporine positive control. Bars display means and SEM from triplicate cultures in one of three experiments. ** P<0.01 different from medium control.

Both parental and experimental strains of *L. jensenii* 1153 colonized morphologically intact epithelial cell monolayer observed by light microscopy at the end of each time period. Transmission electron microscopic images were obtained 24 h post colonization (Figure 1a). The lack of bacteria-induced apoptosis in our model was confirmed by assessment of cleaved versus total caspase 3, showing significant increases of cleaved caspase 3 only by the staurosporine control (Figure 1b).

**Figure 2:**
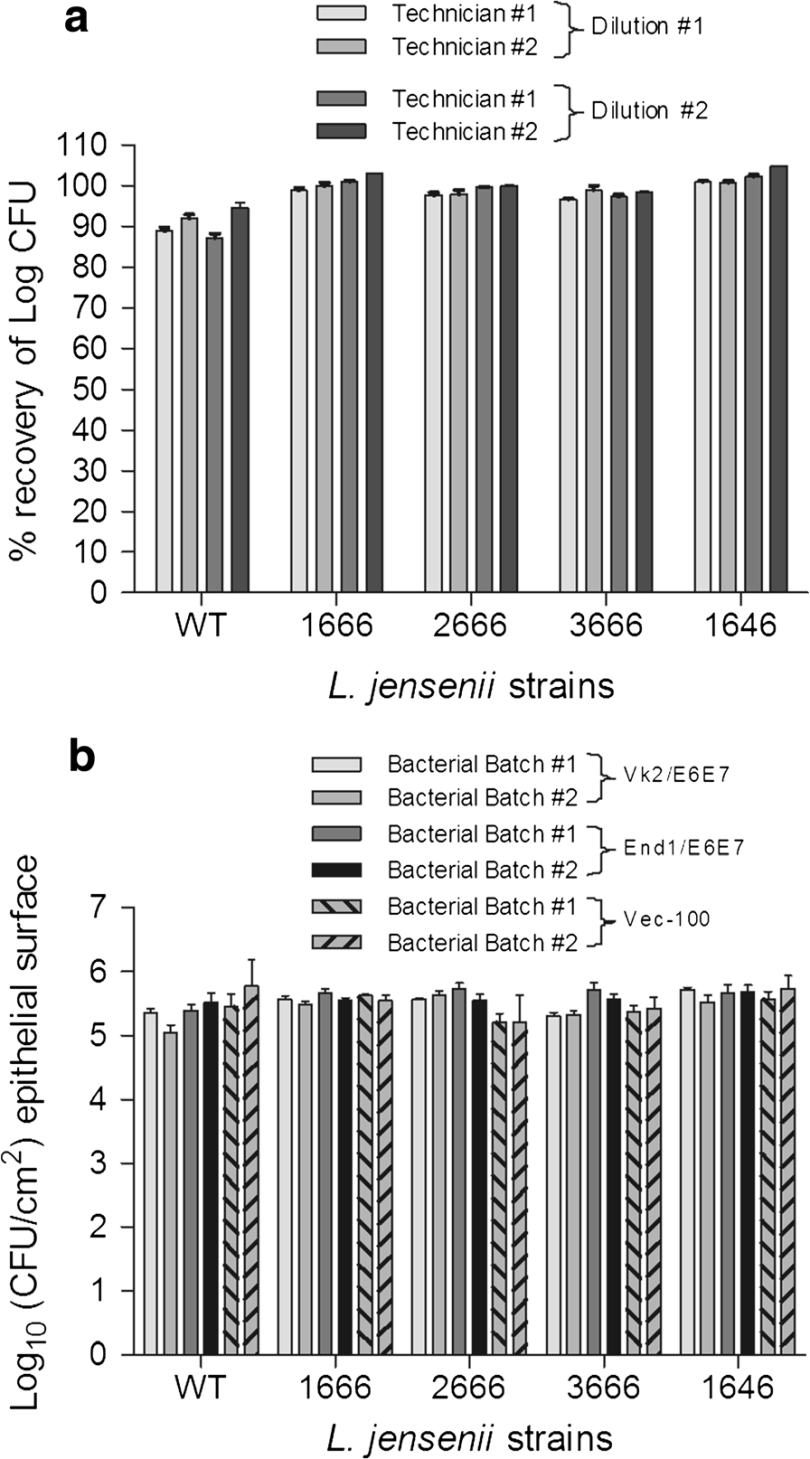
**Technical standardization elicits reproducible results in colony forming units. ***L. jensenii* 1153 wild type (WT) and bioengineered *L. jensenii* 1153–1666, 2666, 3666 and 1646 strains before and after coculture with vaginal and cervical epithelia. (Figure 2a) Full recovery from frozen bacterial stocks and technical reproducibility assessed by % recovery of log CFU in multiple dilutions of quadruplicate cultures prior to epithelial coculture. (Figure 2b) *L. jensenii* strains at 7x10^6^ CFU/ml colonize vaginal (Vk2/E6E7), primary (VEC-100™) and immortalized (End1/E6E7) cervical epithelia at a consistent rate in two separate batches of multiple experiments. Bars represent mean and SEM of triplicate or quadruplicate cultures.

All *L. jensenii* strains demonstrated reproducible recovery from frozen bacterial stocks measured by CFU. No variation was found due to performing technicians or dilutions in multiple bacteria batches tested (Figure 2a).

Wild type *L. jensenii* and all bioengineered derivatives reproducibly generated similar epithelial cell associated CFU counts. Comparable results were obtained with the primary polarized/stratified VEC-100 tissue model as with the immortalized cervical and vaginal epithelial monolayer models. These results were confirmed by comparable colonization rates in multiple experiments with two separate batches of WT and bioengineered bacteria (Figure 2b).

### Wild type and bioengineered *L. jensenii* strains induced NF-κB activation but not proinflammatory protein production

**Figure 3:**
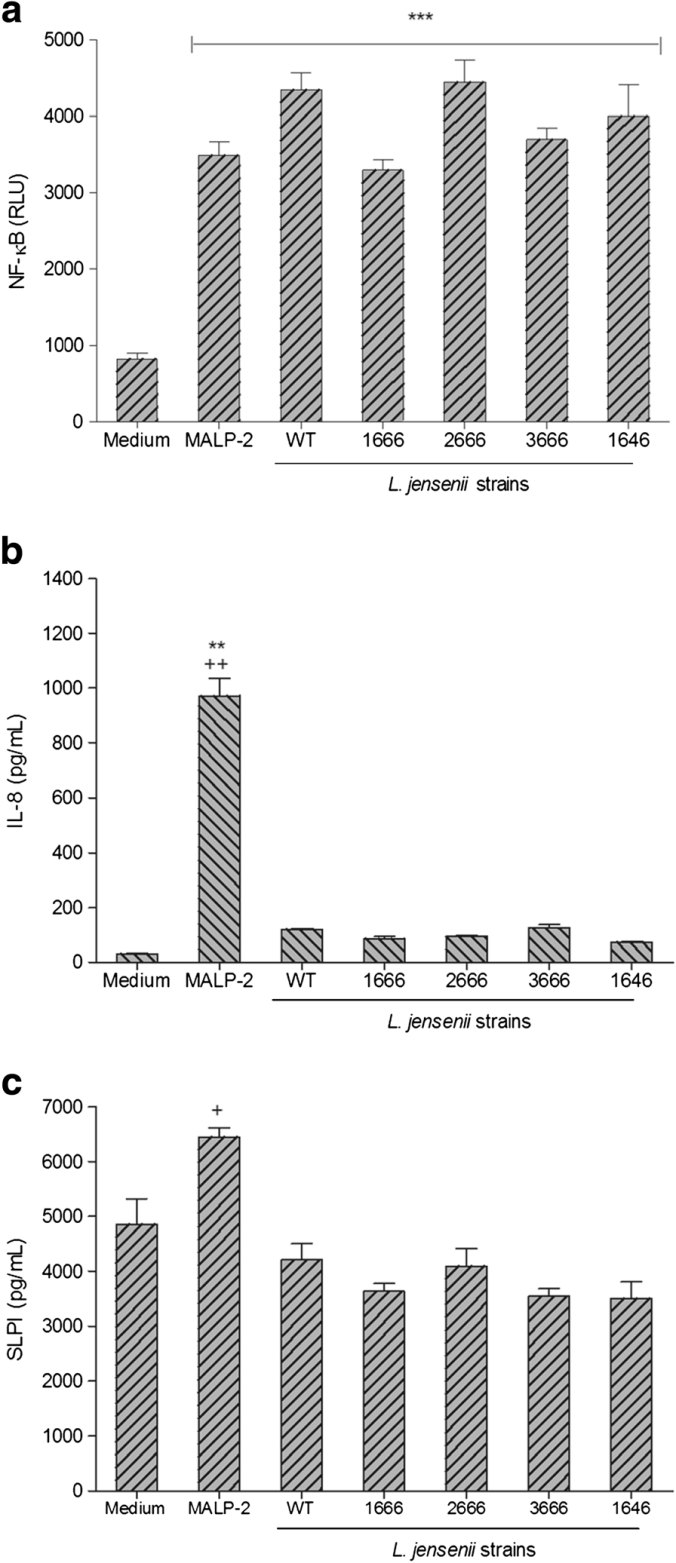
***L. jensenii *induced NF-κB expression without immunogenic response.** 24 h lysates and supernatants harvested from endocervical (End1/E6E7) epithelial cells cultured with 7x10^6^*L. jensenii* 1153 wild type (WT), bioengineered *L. jensenii* 1153–1666, 2666, 3666 and 1646 strains or MALP-2 (50 nM) as a positive control. (Figure 3a) Luciferase activity measured in lysates from triplicate cultures in one representative of five experiments. Bars represent means and SEM ***P<0.001 different from medium control. (Figure 3b) IL-8 production analyzed in corresponding supernatants, bars are means and SEM from duplicate cultures in one representative of 11 experiments **P<0.01 different from medium control, ^*++*^*P*<0.01 different from *L. jensenii* WT. (Figure 3c) SLPI detected in the same supernatants, bars are mean and SEM of duplicate cultures in one representative of six experiments ^*+*^*P*<0.05 different from *L. jensenii* WT.

In order to compare the proinflammatory potential of the WT and derivative bacterial strains, we first examined their effects on the endocervical epithelial cell line stably transfected with the NF-κB-driven luciferase reporter gene in the first 24 h of bacterial-epithelial coculture. Luciferase was measured in cell lysates and IL-8 and SLPI were measured in the paired cell culture supernatants from the same cultures. All bacterial strains caused NF-κB driven luciferase activity similar to that induced by the TLR2/6 ligand MALP-2 (Figure 3a) at significantly (*P<0.001*) higher levels than the sterile medium control (~4-fold increase). However, only MALP-2 induced a significant (*P*<0.01) IL-8 increase (>30-fold) as compared to the medium (no bacteria) control (Figure 3b). MALP-2 alone induced a significant (*P*<0.05) although moderate (<2-fold) increase in SLPI levels measured in the same endocervical cultures as compared to the WT *L. jensenii* (Figure 3c). IL-8 and SLPI levels were not significantly changed by colonization with both the WT and mCV-N expressing bacteria as compared to medium control.

**Figure 4:**
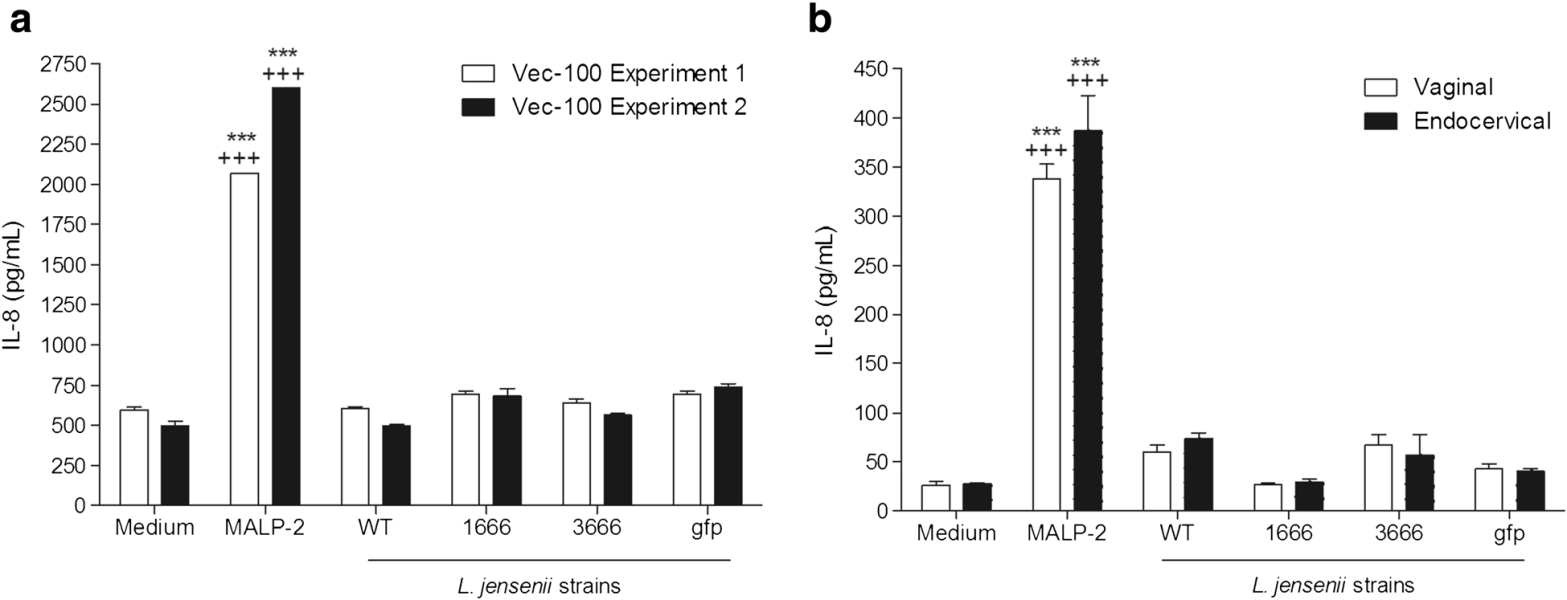
**Cytokine profiles induced by bacteria or synthetic TLR2/6 ligand in cervicovaginal colonized epithelial model.** Similar IL-8 levels measured in supernatants derived from primary and immortalized epithelial cells cultured with *L. jensenii* 1153*–*1666, 3666, gfp bioengineered and *L. jensenii* 1153 wild type (WT) strains or MALP-2 50 nM as a positive control. (Figure 4a) Two independent experiments with (VEC-100™) primary ectocervical originated tissue. (Figure 4b) Vaginal (Vk2/E6E7) and endocervical (End1/E6E7) epithelial colonized cells in one representative of three experiments. Bars represent mean and SEM from duplicate cultures. *** P<0.001 different from medium control, ^*+++*^*P*<0.001 different from *L. jensenii* WT.

To confirm these findings in the primary tissue model, we treated VEC-100™, Vk2/E6E7 and End1/E6E7 cells simultaneously with medium, MALP-2, the WT and bioengineered *L. jensenii* derivatives (Figure 4). Again, MALP-2, in contrast to *L. jensenii*, induced a significant IL-8 upregulation in all three models. Since the findings in the primary tissue model (Figure 4a) mirrored those in the immortalized epithelial monolayers (Figure 3b and 4b), as previously reported with other vaginal bacteria [20], we chose the immortalized cell line model for further analysis of immunity mediators and CFU counts based on its lower cost- and handling time efficiency.

**Figure 5:**
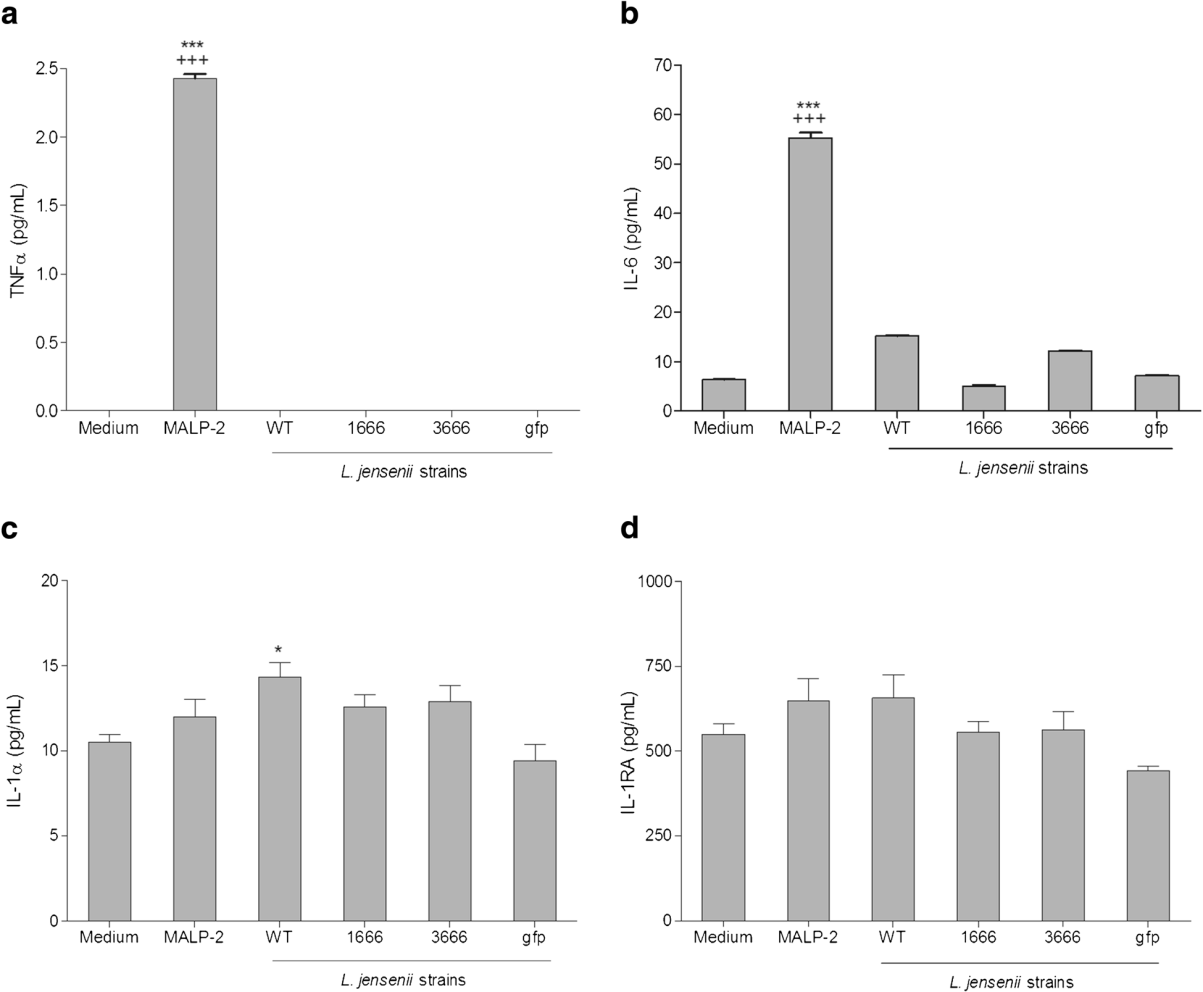
**Absence of a pro-inflammatory cytokine response in *L. jensenii *colonized epithelial model.** (Figure 5a) TNF-α, (Figure 5b) IL-6, (Figure 5c) IL-1α, (Figure 5d) IL-1RA cytokine levels measured in supernatants from vaginal (Vk2/E6E7) epithelium cultured for 24 h with *L. jensenii* 1153*–*1666, 3666, and gfp bioengineered strains and *L. jensenii* 1153 wild (WT) strain or MALP-2 (50 nM) as a positive control. Bars represent mean and SEM from duplicate and triplicate cultures in two independent experiments. *** P<0.001,* P<0.05 different from medium control, ^*+++*^*P*<0.001 different from *L. jensenii* 1153 WT.

In further immune mediator analysis of *L. jensenii* colonized Vk2/E6E7 immortalized epithelial monolayers; MALP-2 induced significant increases over baseline levels of TNF-α (*P*<0.001) and IL-6 (*P*<0.001), while the WT and derivatives had no significant effect on either (Figure 5a-b). IL-1α levels slightly increased (*P*<0.05) in the presence of the WT, however all derivatives maintained baseline levels (Figure 5c). No significant differences were observed in IL-1RA levels (Figure 5D).

### Sustained bacterial colonization by wild type and bioengineered *L. jensenii* does not alter levels of inflammation-associated proteins over time

**Figure 6:**
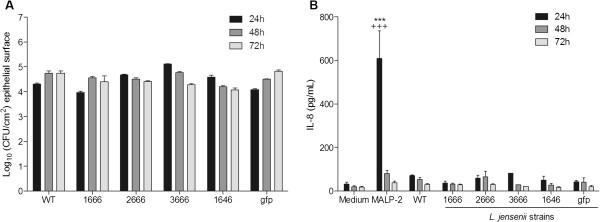
***L. jensenii *consistently colonize epithelial model over a 72 h time period in the absence of IL-8 upregulation.** Vaginal epithelial colonization of *L. jensenii* 1153*–*1666, 2666, 3666, 1646 and gfp bioengineered strains compared with *L. jensenii* 1153 wild type (WT) strain at the end of 24 h, 48 h, and 72 h, time points. (Figure 6a) Colony forming units (CFU) enumerated from lysates harvested at the end of each 24 h incubation time period. (Figure 6b) Consistent IL-8 profile maintained over time measured in the corresponding supernatants collected at the end of each 24 h incubation. Bars represent mean and SEM from duplicate cultures in four independent experiments. ***P<0.001, **P<0.001 different from medium control, ^*+++*^*P*<0.001*,*^*+*^*P*<0.001 different from *L. jensenii* WT.

To determine if the homeostatic effect of *L. jensenii* on innate immunity proteins is sustained over time, despite NF-κB activation, we exposed the vaginal epithelial cells to wild type and bioengineered bacterial strains and MALP-2 and maintained the cultures for three days with supernatants harvested for protein measurement and replaced with plain KSFM medium at each 24 h interval. At the end of each 24 h time period epithelial cells were lysed for assessment of epithelia-associated CFU. No significant variation in CFU was observed in multiple cultures of *L. jensenii-*colonized vaginal epithelial cells over the extended period of 72 h (Figure 6a). The WT and derivatives maintained steady baseline IL-8 levels at 24 h, 48 h, and 72 h with no significant differences observed between the WT and bioengineered bacteria (Figure 6b). As expected, MALP-2 increased IL-8 significantly in the first 24 h time point as compared to both medium control and wild-type colonized bacteria (*P*<0.001), and after its removal at 24 h, the IL-8 levels returned to normal the end of the 72 h period.

**Figure 7:**
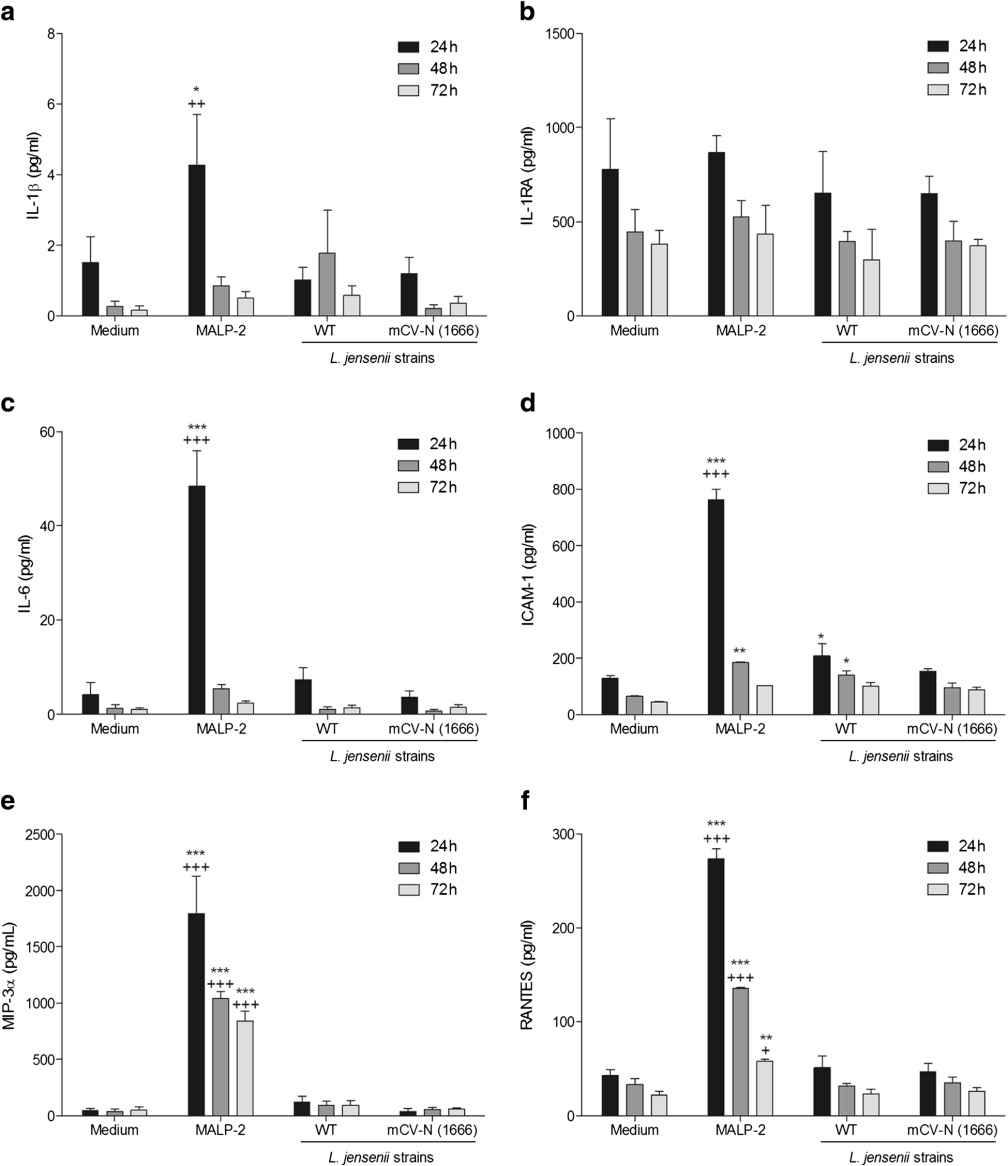
**Bacterial colonization by wild type and bioengineered *L. jensenii*sustained for 72 h does not alter levels of inflammation-associated proteins.** Levels of immune mediators measured in cell culture supernatants by MSD multiplex after colonization of vaginal epithelial cells to by *L. jensenii* 1153 and modified Cyanovirin-N (mCV-N) expressing 1153–1666 for 72 h or after 24 h exposure to MALP-2 and subsequent change of medium at 24 h, 48 h, and 72 h. Bars represent mean and SEM from duplicate cultures in four independent experiments. ***P<0.001, ** P<0.01, * *P*<0.05 different from medium control, ^*+++*^*P*<0.001*,*^*++*^*P*<0.01, ^*+*^*P*<0.05 different from *L. jensenii* WT.

To determine if the lack of proinflammatory protein upregulation over time is a broader phenomenon in the *L. jensenii* colonized vaginal epithelium we expanded our analysis using a multiplex MSD assay to quantify in the same supernatants more mediators known to be associated with the different steps of inflammatory cascades in the female genital tract e.g. pro-inflammatory cytokines IL-1β and IL-6, anti-inflammatory protective mediators e.g. IL-1RA, adhesion molecules e.g. sICAM-1 and chemokines MIP-3α and RANTES. As shown in Figure 7, neither WT nor mCV-N expressing *L. jensenii* induced a significant upregulation or down regulation of any of these mediators with the exception of ICAM-1 which was increased in WT-colonized vaginal cells in the first 48 h only (*p<0.05*) (Figure 7d). In contrast, MALP-2 induced a weak upregulation of IL-1β (*p<0.05*) (Figure 7a), no change in IL-1RA (Figure 7b) but a robust (several-fold) upregulation (*p<0.001*) of IL-6, ICAM-1, MIP-3α and RANTES (Figure 7c-f), and the chemokines remained increased for 48 h after MALP-2 removal (Figure 7e and f).

### Expression of functional mCV-N expression and anti-HIV activity is preserved in epithelia-associated *L. jensenii* strains

**Figure 8:**
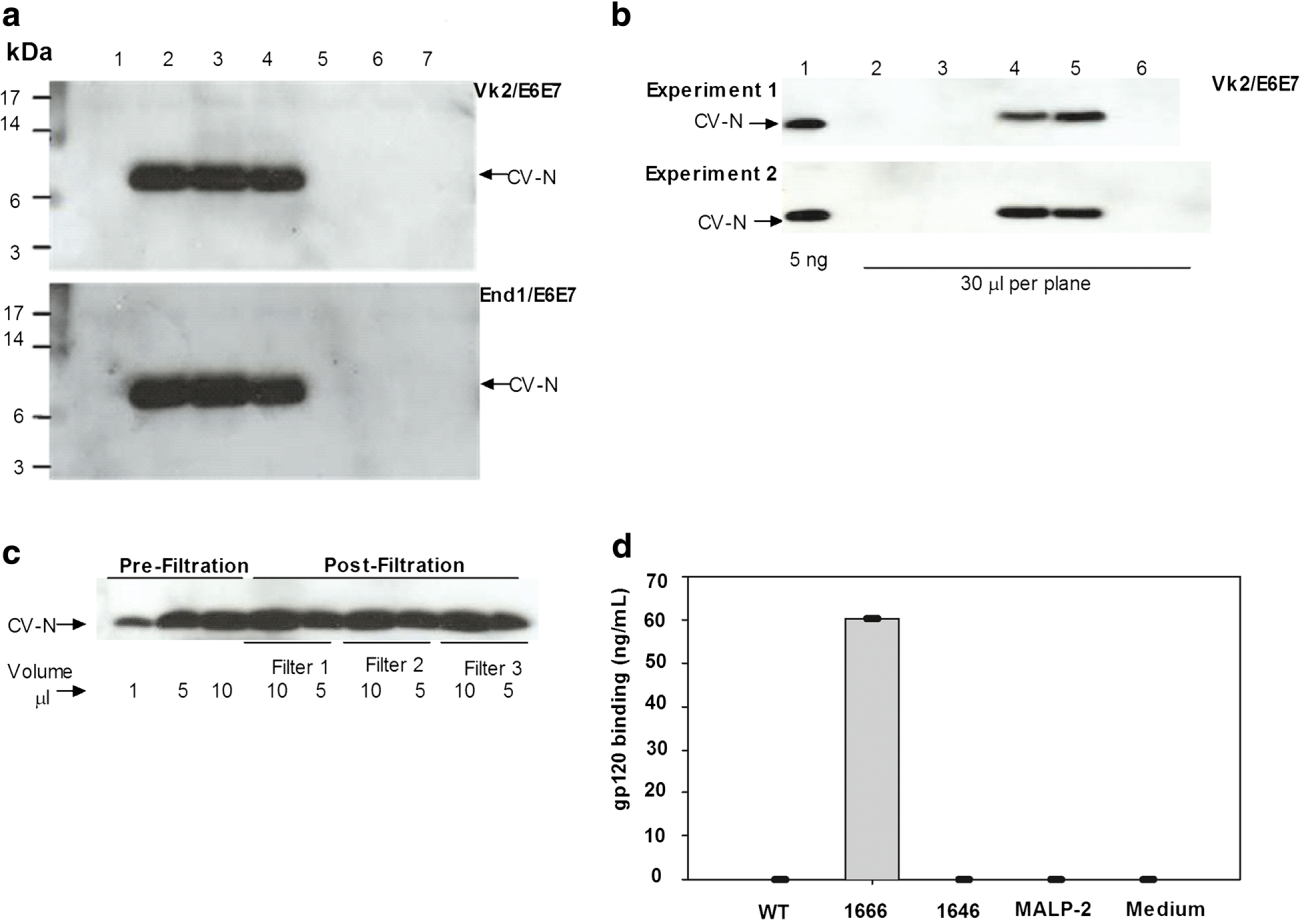
**Epithelial colonized *L. jensenii*preserve potent anti-HIV properties.** Western blot from 24 h sterile supernatants collected from *L. jensenii-*colonized vaginal (Vk2/E6E7) and endocervical (End1E6E7) epithelial cells demonstrate consistent preservation of modified Cyanovirin-N (mCV-N) expression in mCV-N producing strains. (Figure 8a) mCV-N producing bioengineered strains (*L. jensenii* 1153–1666, 2666 and 3666) located in lanes #2, 3 and 4 are contrasted to *L. jensenii* 1153 WT in lane #1, the β-glucuronidase expressing strain *L. jensenii* 1153–1646 in lane #5, MALP-2 control in lane #6, and medium control in lane #7. (Figure 8b) A mCV-N standard in lane #1 is compared to the mCV-N producing *L. jensenii* strains: *L. jensenii* 1153–1666 and 3666 in lanes #4 and #5 in contrast to the green florescent protein expressing strain *L. jensenii* 1153-gfp in lane #6, MALP-2 in lane #3 and medium control in lane #2. (Figure 8c) No loss to filtration is observed in western blot analyses of mCV-N before and after spiking one ml of media with one μg mCV-N. (Figure 8d) gp120 binding activity in one representative mCV-N producing *L. jensenii* 1153–1666 strain detected by a gp120 binding assay in sterile supernatants collected from 24 h *L. jensenii* colonized vaginal (Vk2/E6E7) epithelial culture. Data are from one representing three independent experiments.

Filtered sterile supernatants from 24 h *L. jensenii* colonized vaginal and endocervical cells were assessed for mCV-N recovery with western blot analysis on an SDS-PAGE gel probed with anti-CV-N antibodies. All mCV-N expressing strains (lanes 2–4; Figure 8a, lanes 4–5; Figure 8b) produced full length mCV-N as compared to a mCV-N standard (lane 1; Figure 8b). As expected, no background binding to mCV-N was detected in cell culture supernatants derived from the MALP-2 or medium controls (lanes 6–7; Figure 8a) or from either the WT (lane 1; Figure 8a, lane 2; Figure 8b) or β-glucuronidase producing strains (lane 5; Figure 8a, lane 6; Figure 8b). No protein loss to filtration was observed when 1 μg of mCV-N standard was spiked in 1 ml of medium and probed with anti-mCV-N antibody in a western blot pre and post-filtration (Figure 8c).

Gp120 binding activity was measured in 24 h filtered sterile supernatants from *L. jensenii* colonized cervical and vaginal epithelial cells. Only the mCV-N producing strain resulted in gp120 binding activity compared to the WT and β-glucuronidase producing strains, MALP-2 or medium control (Figure 8d). Data were replicated in multiple experiments not shown here.

## Discussion

Vaginal probiotics or live biotherapeutic products as defined by the FDA [39] may reduce the risk of HIV transmission by: expressing antiviral factors, restoring the normal microbiota, inhibiting bacterial pathogens and modulating immuno-inflammatory responses without compromising the homeostatic environment of the host. Lactobacilli are commensal Gram-positive bacteria that widely populate the healthy female vaginal mucosa [21,22,40,41]. Several *Lactobacillus* strains have been implicated by epidemiologic and/or experimental evidence in the maintenance of a homeostatic infection-free microenvironment most notably due to the impact of the bacteria’s lactic acid and H_2_O_2_ production in generating an adverse environment for HIV and other STDs. [21,40,42-44]. These properties may contribute to the reduction of viral particles at the site of infection [13,45]. In contrast, a reduction in the number of *Lactobacillus* in the vaginal microbiota has been associated with the acquisition of bacterial vaginosis (BV) [42,45-47]. The presence of BV is correlated with an increased risk of acquiring herpes simplex virus type 2 [48], HIV and other STDs [46,49]. In turn, co-infection with sexually transmitted pathogens is associated with an increased risk of acquiring and transmitting HIV [50,51]. Naturally occurring lactobacilli demonstrate an inverse relationship with HIV infectivity [44,45]. Sha *et al.* found an inverse ratio between indigenous *Lactobacillus* counts and HIV RNA detected in cervical vaginal lavage at nearly significant levels [46]. In another study, *L. jensenii* demonstrated a reduction in HIV infection by 23% *in-vitro*[26].

Our finding that *L. jensenii* can induce NF-κB activation and at the same time maintain low levels of inflammation-associated proteins has important implications for its potential use as a vaginal probiotic or biotherapeutic. NF-κB is a major transcription factor that plays a key role in inflammatory disease and upregulates a myriad of inflammation-associated genes including those studied here [52]. At the same time NF-κB participates in its own negative feedback loop promoting the resolution of inflammation *in-vivo*[53]. Thus, the net effect of NF-κB activation depends on the cell and tissue context, the interplay of a number of intra- and extra-cellular factors, and the nature of the activating signal. It has been previously shown that some lactobacillus species (*L. crispatus* and *L. acidophilus*) can cause NF-κB activation and yet maintain low levels of IL-8 and RANTES [20]. Another study showed that *L. jensenii* can suppress IL-8 induced by TLR ligands [54]. Interestingly, a non-vaginal lactobacillus species (*L. kefiranofaciens*) induced production of MIP-3α [55] and other vaginal bacteria, associated with bacterial vaginosis e.g. *P. bivia* and *A. vaginae* induced simultaneous NF-κB activation and upregulation of inflammatory proteins in contrast to vaginal *L. crispatus* and *L. acidophilus*, which maintained low levels of proinflammatory proteins in the vaginal colonization context [20]. We now demonstrate for the first time using an expanded panel of innate immunity mediators that this immuno-modulatory phenomenon is also true for the *L. jensenii* isolate 1153 and its bioengineered derivatives. The results of our study agree with clinical observations showing an association of vaginal lactobacilli with relatively low levels of pro-inflammatory mediators *in-vivo*[56-58]. Furthermore, the results from our *in-vitro* model are in agreement with findings generated in a macaque model of SHIV infection [26]. Vaginal levels of IL-6, IL-8, IL-1β and IL-1RA were not different between macaques with no lactobacilli, those colonized with lactobacillus indigenous for the macaque and those colonized with mCV-N expressing *L. jensenii* 1153–1666 [26]. Other commensal bacteria have also been shown to downregulate inflammatory responses. For example, *H. pylori* downregulated IL-8, MIP-3α and other chemokines through inducing microRNA expression in host epithelial cells [59]. Further research is required to determine the molecular mechanisms, by which vaginal *L. jensenii*, *L. crispatus* and *L. acidohilus* tune the host innate immune responses to avoid proinflammatory protein production in the presence of a potent NF-κB activation*.*

The innate immunity mediators assessed here (TNF-α, IL-1α, IL-1RA, IL-6, ICAM-1, IL-8, RANTES, MIP-3α and SLPI) are known as indicators of mucosal toxicity, and inflammation and have been used and recommended for microbicide safety evaluation [32,35,60]. In contrast to IL-1RA, which displays anti-inflammatory properties [35,61], the pro-inflammatory cytokines IL-1α, TNF-α, IL-6 and IL-8 can activate HIV viral replication in infected cells [62-66]. Similarly vaginal inflammation increases the risk of HIV transmission by increasing the number of host cells at the site of infection [35,67,68]. IL-8 is also involved in the recruitment of innate immune cells, neutrophils and CD4 positive T-cells to the site of infection [32,64,69]. MIP-3α is a chemokine recruiting dendritic cells and along with RANTES, a chemokine for T cells, is known to play a role in the early recruitment of HIV target cells [70,71]. Thus, the lack of upregulation of these proinflammatory mediators by the cervicovaginal epithelial cells is a desired safety feature of the mCV-N expressing *L. jensenii* strain. Concerns about the safety of CV-N in the absence of lactobacillus have been raised by Huskens *et al*. [72] showing that administration of CV-N to pre-stimulated PBMC induced proinflammatory cytokine upregulation and it also had *in-vitro* mitogenic activity. It is important to clarify that the study by Huskens *et al*. is of limited relevance to the clinical application of the mCV-N-expressing lactobacilli for several reasons: 1) the mCV-N is a genetically modified stable monomeric derivative of the natural cyanobacterium-produced CV-N protein referred to in that older study, 2) Huskens *et al*. seemed to have used *E. coli* expressed CV-N protein; however, they don’t address steps taken to eliminate or control for endotoxin contamination in their experiments. In contrast, in our study mCV-N is expressed in the context of lactobacillus which lacks endotoxin.

IL-1α, IL-1RA and SLPI are stored in the epithelial cell and released upon membrane damage [35,61,73]. The fact that none of the *L. jensenii* strains caused significant increase in these mediators suggests preserved membrane integrity in addition to lack of immunotoxicity. A decrease in SLPI levels is also often associated with an increased risk of HIV infection [74,75]. This in addition to the lack of apoptosis assessed by caspase-3 levels suggests that *L. jensenii* is capable of colonizing and self-sustaining the human vaginal epithelia without cellular toxicity. In this model *L. jensenii* produced full-length biologically active mCV-N within the epithelial context. mCV-N did not compromise cell viability or elicit an immuno-inflammatory response when tested in both rabbits and macaques [23,76].

This study confirmed the ability of bioengineered *L. jensenii* strains to reproducibly colonize the cervicovaginal epithelial model and to maintain anti-HIV expression of functional peptides *in-vitro* without the induction of a significant change in inflammation associated proteins. The ability for endogenous lactobacilli to colonize and establish dominance in the vaginal microenvironment has been previously investigated. *Lactobacillus* isolates were successfully introduced intravaginally as a probiotic against BV and urinary tract infections in women [77,78]. In a study conducted by Hemmerling *et al. L. crispatus* colonized BV infected women 61-78% of the time [79]. We found all *L. jensenii* strains including the mCV-N expressing *L. jensenii* (1153–1666) capable of reproducibly and stably colonizing the human cervicovaginal epithelial cells over a 72 h period without significant perturbations to innate immune barrier parameters while abundantly expressing mCV-N detectable by both Western blot and the functional gp120 assay. The stable colonization mCV-N expressing *L. jensenii* 1153–1666 strain and the stability and anti-HIV activity of the mCV-N protein have been confirmed in a mouse model over a period of six days [15] and in the Rhesus macaque for six weeks post inoculation [26], where it reduced SHIV infection by 63% in a repeated challenge model, without altering markers associated with mucosal barrier function. Taken together these *in-vivo* findings provide validation of our *in-vitro* model.

The bioengineered mCV-N, similarly to the natural protein, is stable at a broad pH range from 4–8.2 [15,23]. This wide pH stability spectrum encompasses both the acidic pH generated by lactic acid producing bacteria and the slightly more alkaline pH introduced to the vaginal environment with seminal fluid. The natural and modified CV-N molecules are also resistant to thermal and chemical denaturation, which would allow it to be produced and stored in a variety of environmental conditions [15,23]. These attributes render mCV-N to be a promising microbicide candidate.

In this proof-of-concept *in-vitro* model, the bioengineered *L. jensenii* did not differ from the wild type parental strain in term of epithelial colonization capacity and did not induce a pro-inflammatory profile in the human epithelial cell context. Thus, our *in-vitro* findings along with *in-vivo* studies performed in the murine and macaque model pave the way to further clinical safety evaluations necessary to confirm the effects these bacteria would have when introduced into the human cervicovaginal environment and how it would affect other endogenous microbiota *in-vivo*. There are many components that are unique to the human vaginal environment and therefore would be best investigated *in-vivo* i.e. indigenous bacterial biofilms, pH, mucosal immunoglobulins and hormones, and vaginal practices that may modify the effects of both the bioengineered bacteria and the activity of mCV-N peptide.

## Conclusion

Our *in-vitro* human vaginal colonization model produced consistent results, validated by their agreement with findings from the *in-vivo* macaque model. Because of its reproducibility and low cost, the *in-vitro* colonization model can be used for high throughput preclinical screening and side-by-side comparison of multiple bacterial strains, bioengineered derivatives and probiotic candidates to select those with best homeostatic properties. In support of our hypothesis, we were able to compare microbiota-epithelial interactions of multiple *L. jensenii* WT and bioengineered strains in a reproducible manner. The bioengineered *L. jensenii* derivatives were able to deliver a bioactive anti-HIV peptide without inducing cellular toxicity or alterations in levels of pro-inflammatory cytokines and protective mucosal immune mediators e.g. SLPI or IL-1RA. Our pre-clinical safety data in combination with the results from the macaque model provide support for future clinical evaluations of the bioengineered *L. jensenii* bacteria as an anti-HIV microbicide.

## Competing interests

QX was previously employed by Osel, Mountain View, CA, the company that has provided the bioengineered strains for this study.

## Authors’ contributions

HSY wrote the manuscript, ran the immunoassays and conducted the experiments along with RNF. RNF was responsible for the direction of the study, experimental design and data integrity. QX provided all bacterial strains and bioengineered derivatives, directed the western blot and gp120 binding assays, reviewed the progress and manuscript, and provided comments. All authors read and approved the final manuscript.
